# Complete Genome Sequence of Phascolarctobacterium faecium JCM 30894, a Succinate-Utilizing Bacterium Isolated from Human Feces

**DOI:** 10.1128/MRA.01487-18

**Published:** 2019-01-17

**Authors:** Yusuke Ogata, Wataru Suda, Nao Ikeyama, Masahira Hattori, Moriya Ohkuma, Mitsuo Sakamoto

**Affiliations:** a Laboratory for Microbiome Sciences, RIKEN Center for Integrative Medical Sciences, Yokohama, Japan; b Graduate School of Advanced Science and Engineering, Waseda University, Tokyo, Japan; c Microbe Division/Japan Collection of Microorganisms, RIKEN BioResource Research Center, Tsukuba, Ibaraki, Japan; d PRIME, Japan Agency for Medical Research and Development (AMED), Tsukuba, Ibaraki, Japan; University of Rochester School of Medicine and Dentistry

## Abstract

Phascolarctobacterium faecium is an anaerobic microbe known as a member of the human gut microbiome. Here, we report the complete genome sequence of Phascolarctobacterium faecium JCM 30894 and the elucidation of the mechanism for utilization of succinate by this bacterium based on the genome analysis.

## ANNOUNCEMENT

Phascolarctobacterium faecium is an anaerobic and Gram-negative bacterium that was first isolated from koala feces (1). P. faecium is difficult to cultivate because the metabolic requirements for growth are unclear. However, it was reported that Phascolarctobacterium succinatutens, a species similar to P. faecium, utilized succinate to grow well under the conditions supplemented with succinate (2). We successfully cultivated P. faecium JCM 30894, which was isolated from a fecal sample from a healthy Japanese man (46 years old), with the Gifu anaerobic medium (Nissui) supplemented with 1% (wt/vol) succinate for 4 days at 37°C under a H_2_/CO_2_/N_2_ (1:1:8, by volume) gas mixture, which allowed us to sequence a genome of this strain.

The genomic DNA of P. faecium JCM 30894 was prepared according to the method previously reported (3). Briefly, bacterial cells were incubated with 15 mg/ml lysozyme and 2,000 unit/ml achromopeptidase, and then treated with 1% sodium dodecyl sulfate (SDS) and 1 mg/ml proteinase K. The genomic DNA was extracted with phenol-chloroform–isoamyl alcohol, isolated by ethanol precipitation, and purified by RNaseA treatment, followed by polyethylene glycol 6000 (PEG 6000) precipitation. The sequencing was performed with the Illumina MiSeq and the PacBio Sequel platforms. For the Illumina sequencing, the library was prepared using TruSeq DNA PCR-free library prep kit (Illumina), and the target insert size was 550 bp. The Illumina paired-end sequencing yielded 5,042,362,254 bases from 8,543,389 reads, with an average length of 291 bp. All the Illumina reads were trimmed and filtered using FASTX-toolkit (v. 0.0.13; http://hannonlab.cshl.edu/fastx_toolkit/), and then the human genome and internal control were removed by mapping with Minimap2 (v. 2.13-r850) (4). For the PacBio sequencing, the library was prepared using the SMRTbell template prep kit 1.0, and the single-read sequencing yielded 910,454,478 bases from 369,987 reads, with an average length of 2,460.8 bp, and then human genome and internal control were removed by mapping with Minimap2. As a result of trimming, filtering, and removing controls, the Illumina sequencing yielded 1,790,703,092 bases from 3,241,535 pair-end reads, with an average length of 279.1 bp, and the PacBio sequencing yielded 434,445,832 bases from 178,532 reads, with an average length of 2,435.9 bp. Both reads were assembled using the hybrid assembler Unicycler (5) to obtain the complete circular single contig with an average read depth of 1,076×. The genes were predicted using Prokka (v. 1.11) (6) and analyzed by using Kyoto Encyclopedia of Genes and Genomes (KEGG; release 63.0) (7), Rapid Annotations using Subsystem Technology (RAST; v. 2.0) (8), and DDBJ Fast Annotation and Submission Tool (DFAST; v. 1.0.2) (9). The default settings were used.

The P. faecium JCM 30894 genome comprised a circular chromosome of 2,454,371 bp, with a G+C content of 43.70%, containing 2,335 protein-coding genes with 231 tRNA and 20 rRNA genes. The genome analysis revealed that this genome contained two pathways for energy production. One was the production of one propionate and one ATP from one phosphoenolpyruvate and one ADP, similar to an anaerobic bacterium, Bacteroides fragilis (10). Another was the production of one propionate and two ATPs from one phosphoenolpyruvate, one orthophosphate, and two ADPs. However, the P. faecium JCM 30894 genome lacked fumarate reductase which was an enzyme necessary to produce succinate converted to propionate in the both pathways. This enzyme was also lacking in the P. succinatutens genome. Thus, our data suggest that additive succinate serves as a key material in both energy production pathways for the growth of P. faecium JCM 30894.

### Data availability.

The complete genome sequence of P. faecium JCM 30894 was deposited in DDBJ/ENA/GenBank under the accession no. AP019004, which is linked to the BioProject accession no. PRJDB7520.
